# Proteolytic cleavage of stingray phospholipase A2: Isolation and biochemical characterization of an active N-terminal form

**DOI:** 10.1186/1476-511X-10-124

**Published:** 2011-07-26

**Authors:** Abir G Ben Bacha, Hafedh Mejdoub

**Affiliations:** 1 Biochemistry Department, Science College, King Saud University, P.O box 22452, Zip code 11495, Riyadh, Saudi Arabia; 2 Laboratoire des biotechnologies végétales appliquées à l'amélioration des cultures, Faculty of Science of Sfax, Sfax University, 3038 Sfax, Tunisia

## Abstract

**Background:**

Mammalian GIB-PLA2 are well characterized. In contrast, much less is known about aquatic ones. The aquatic world contains a wide variety of living species and, hence represents a great potential for discovering new lipolytic enzymes. The aim of this study was to check some biochemical and structural properties of a marine stingray phospholipase A2 (SPLA2).

**Results:**

The effect of some proteolytic enzymes on SPLA2 was checked. Chymotrypsin and trypsin were able to hydrolyze SPLA2 in different ways. In both cases, only N-terminal fragments were accumulated during the hydrolysis, whereas no C-terminal fragment was obtained in either case. Tryptic and chymotryptic attack generated 13 kDa and 12 kDa forms of SPLA2, respectively. Interestingly, the SPLA2 13 kDa form was inactive, whereas the SPLA2 12 kDa form conserved almost its full phospholipase activity. In the absence of bile slats both native and 12kDa SPLA2 failed to catalyse the hydrolysis of PC emulsion. When bile salts were pre-incubated with the substrate, the native kinetic protein remained linear for more than 25 min, whereas the 12 kDa form activity was found to decrease rapidly. Furthermore, The SPLA2 activity was dependent on Ca^2+^; other cations (Mg^2+^, Mn^2+^, Cd^2+ ^and Zn^2+^) reduced the enzymatic activity notably, suggesting that the arrangement of the catalytic site presents an exclusive structure for Ca^2+^.

**Conclusions:**

Although marine and mammal pancreatic PLA2 share a high amino acid sequence homology, polyclonal antibodies directed against SPLA2 failed to recognize mammal PLA2 like the dromedary pancreatic one. Further investigations are needed to identify key residues involved in substrate recognition responsible for biochemical differences between the 2 classes of phospholipases.

## 1. Introduction

Phopholipases A2 (PLA2s) are esterases that catalyse the hydrolysis of acyl groups at the sn-2 position of glycero-phospholipids (PL) and produce free fatty acids, such as arachidonic acid, and lyso-PL by an interfacial activation catalytic mechanism [1].

A large number of distinct PLA2s have been characterized and classified into the broad categories of intracellular and secreted forms of the enzyme. Intracellular (cytosolic) PLA2s participate in cellular eicosanoid metabolism and signal transduction. Numerous isoforms of secretory phospholipases (sPLA2s) have been identified and divided into several groups, based on their amino acid sequences, structures, catalytic mechanisms, tissue distributions and evolutionary relationships [1,2]. Ten human secreted PLA2s have been cloned: group (G) IB, GIIA, GIID, GIIE, GIIF, GIII, GV, GX, and GXIIA PLA2s and the GXII PLA2-like protein that is devoid of catalytic activity [3] and [4]. sPLA2s are low molecular weight enzymes (14 kDa) and are abundant in various mammalian tissues and fluids. These enzymes are involved in various normal and pathological cell functions [1]. A Group IB enzyme was first found in mammalian pancreatic exudates, and therefore is known as pancreatic PLA2. The main physiologic function of GIB PLA2 is to digest dietary phospholipids. GIB PLA2 is synthesized in pancreatic acinar cells and secreted into the duodenal lumen as an enzymatically inactive PLA2 that is activated by cleavage of a 7-amino acid activation peptide by trypsin [5].

The sPLA_2_s from mammalian pancreas and snake venoms have been used as diagnostic biochemical reagents. In fact snake venom sPLA_2_s were employed to analyse the position of fatty acids in phospholipids from guina pig and pic cardiac membranes [6]. Moreover, porcine PLA_2 _(PPLA2) was used in the examination of the structural and functional changes of egg yolk low density lipoproteins (LDL) by modifying its phospholipids [7]. In addition, PPLA_2 _was used in industrial processes in the food industry to produce lyso PC which is an excellent emulsifier for food [8]. The biology and the biochemistry of mammalian and venom PLA_2 _are well documented. In contrast, few studies were reported on the enzymology and application of PLA_2 _from marine organisms [9]. Therefore, little information is available on marine gastropod mollusc's sPLA_2 _[10-16]. To date, few studies exist on phospholipase from the digestive gland of marine organisms. Recently, we have purified stingray PLA2 (SPLA2) from the common stingray *Dasyatis pastinaca *and some of its catalytic properties were determined [17]. High similarity was found between the N-terminal amino acid residues of SPLA2 and those of other known pancreatic PLA2. In the presence of organic solvents, as well as in acidic and alkaline pH and at high temperature, SPLA2 stability makes it a good candidate for its application in food industry. It seems therefore of interest to check some other catalytic and structural properties of SPLA2 to gain more insights into their action mode on phospholipids. We have therefore performed the limited proteolysis experiments on SPLA2, using trypsin and chymotrypsin. Profiles regarding proteolysis and activity are reported.

## 2. Material and methods

### 2.1. Proteins

SPLA2 and DrPLA2 were purified as described by Ben Bacha *et al*., [17,18]. Protein concentration was determined as described by Bradford et al. [19] using BSA (E = 6.7) as reference.

### 2.2. Determination of phospholipase activity

The stingray PLA2 activity was measured titrimetrically at pH 8.5 and at 40°C with a pH-stat, under the optimum conditions, using purified egg phosphatidylcholine (PC), phosphatidylserine (PS) or phosphatidylethanolamine (PE) emulsions as substrate in the presence of 4 mM NaTDC and 8 mM CaCl2 [20]. One unit of phospholipase activity was defined as 1 μmole of fatty acid liberated under standard conditions.

### 2.3. Limited proteolysis

SPLA2 (1 mg) was dissolved in 1 ml of 50 mM Tris-HCl buffer, pH 8.5 without benzamidine. The PLA2 solution was digested at 4, 30 and 37°C with the selected endopeptidase. The endopeptidase/PLA2 molar ratio varied from 0.01 to 0.1. Samples (50 μl) were withdrawn from the incubation mixture at various times to assess the residual activity and the electrophoretic profile. The reaction was stopped by addition of benzamidine (4 mM final concentration).

### 2.4. Analytical methods

Analytical polyacrylamide gel electrophoresis of proteins in the presence of sodium dodecyl sulfate (SDS-PAGE) was performed by the method of Laemmli [21]. Samples for sequencing or immunoblotting were electrotransferred onto polyvinylidene difluorid and a nitrocellulose membrane respectively [22]. Protein transfer was performed during 2 h at 4 mA/cm^2 ^at room temperature.

### 2.5. Amino acid sequencing

The N-terminal sequence was determined by automated Edman's degradation, using an Applied Biosystems Protein Sequencer Procise 492 equipped with 140 C HPLC system [23].

### 2.6. Production of polyclonal antibodies

Polyclonal antibodies directed against purified SPLA2 were produced on rabbits after subcutaneous and intra-muscular injections every 3 weeks of 0.5 mg of pure SPLA2. The first injection included complete Freund's adjuvant, while the last two injections contained incomplete adjuvant.

### 2.7. Immunoblotting technique

The reactivity of anti-SPLA2 serum with PLA2 (SPLA2 or DrPLA2) was checked using immunoblotting technique. After protein transfer, membranes were rinsed three times with PBS (phosphate buffer saline: 10 mM phosphate pH 7.2, 150 mM NaCl), then saturated with 3% of milk powder in PBS (saturating buffer) for 1 h at room temperature. Thereafter, anti-SPLA2 serum diluted at 1:1000 with PBS containing 0.05% Tween-20 (PBS/Tween-20) were incubated with the membranes for 1 h at room temperature. Afterwards, membranes were washed three times with PBS/Tween-20 then incubated for 1 h at room temperature with a 1:2000 dilution of alkaline phosphatase-conjugated anti-rabbit immunoglobulin (Sigma). After washing, as mentioned above, membranes were incubated with a phosphatase substrate solution containing 0.3 mg/ml of nitroblue tetrazolium chloride (Sigma), 0.2 mg/ml of 5-bromo-4-chloro-3 indolyl-phosphate (Sigma) and 0.2 mg/ml of MgCl_2 _to reveal the specific immunoreactivity.

### 2.8. Enzyme linked immunosorbent assay (ELISA) analysis

The immunoreactivity of anti-SPLA2 polyclonal antibodies with phospholipases (SPLA2 or DrPLA2) was checked, using the ELISA technique. Purified phospholipases (SPLA2 or DrPLA2) were diluted using coating buffer (PBS) to obtain a final concentration of 1 μg/ml. Aliquots (100 μl) were coated onto polyvinyl chloride microtiter wells and incubated overnight at 4°C. The wells were then saturated by adding 100 μl of saturating buffer (3% of powder milk in PBS) for 2 h at 37°C. Thereafter, 100 μl of serum, diluted at 1:500 with saturating buffer, were added to each well and the plates were incubated for 1 h at 37°C. Afterwards, 100 μl of peroxidase-conjugated anti-rabbit immunoglobulin (Sigma) diluted at 1:2000 with saturating buffer were added to each well and the plates were kept at 37°C for an additional hour. Then, 100 μl of freshly prepared peroxidase substrate solution (an o-phenylenediamine tablet (Sigma) was solubilized in 50 mM sodium phosphate/citrate, pH 5 containing 0.4% of fresh hydrogen peroxide) were added to each well. The plates were incubated in the dark for 30 min at room temperature. The enzymatic reaction was then stopped by adding 50 μl of 0.5 M H_2_SO_4_. The absorbance was read at 490 nm in a micro-ELISA reader (Dynatech).

## 3. Results and discussion

### 3.1. Level of expression of SPLA2 activity

In order to compare the level of stingray PLA2 activity with other species, the rate of hydrolysis of PC emulsion by dromedary, bovine, chicken, and turkey pancreases were measured under the same conditions. Results reported in Table 1 show that the pancreases tested secreted various levels of PLA2 activity. The highest level was observed with turkey (200 U/g). Sheep presented only 10 U/g in its pancreas.

**Table 1:** Pancreatic PLA2 levels in some animals

Species	PLA2 activity (U/g fresh pancreas)	References
Stingray	55 ± 5	Present study

Dromedary	20 ± 3.5	[18]

Turkey	200 ± 20	[18]

Bovine	110 ± 17	[18]

Chicken	70 ± 25	[18]

Sheep	10 ± 5	[18]

The determination of the phospholipase content of pancreases from all species was performed in a homogenate prepared in a Waring Blendor (2 × 30 s) with 10 ml, per gram of fresh tissue, of 10 mM Tris-HCl, 150 mM NaCl, and pH 8.5 at room temperature. After centrifugation at 12.000 rpm during 20 min, the amount of enzyme was estimated on an aliquot of the supernatant using PC emulsion as substrate in the presence of 4 mM NaTDC and 8 mM CaCl2. The phospholipase activity was measured titrimetrically at pH 8.0 and at 37°C using a pH-stat. One phospholipase unit corresponds to one μmole of fatty acid released per min using PC emulsion as substrate in the presence of 4 mM NaTDC and 8 mM CaCl2. For each species, the activity represents the average ± standard error mean of five assays obtained from three different pancreases.

### 3.2. Annual distribution of the SPLA2 activity levels

**Figure 1:**
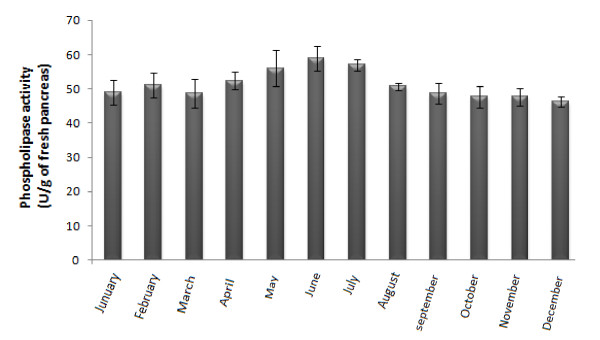
**Phospholipase activity levels found in fresh stangray pancreas during one year**. Activity was measured in optimal conditions using PC as substrate and expressed as the number of units per corporal weight. The bars indicate the standard error of the means of three experiments.

Dasyatis pastinaca used in this study were collected every month between January and December and the SPLA2 activity was observed monthly. As we can see from Figure 1, no significant difference in the PLA2 activity was observed in the monthly collected samples. A slight increase in phospholipase activity was measured during the summer (May to July). This increase can be related to the reproduction activity during this period and to the increase of the temperature of the sea water. Then, Ismen (2003) [24], indicated that this species presented a high reproduction activity from May to September. Moreover, Spawning occurred mainly from May to September, apparently triggered by the rising seawater temperature during summer.

### 3.3. Effect of metal ions on SPLA2 activity

The SPLA2 showed a strict dependence on calcium ions (8 mM) for full activity using PC as substrate. The addition of Mg^2+^, Mn^2+^, Cd^2+ ^and Zn^2+ ^(10 mM) in the absence or presence of low Ca^2+ ^concentration (1 mM) significantly decreased the enzyme activity (Table 2). The obtained data showed that substitution of Ca^2+ ^by other divalent ions (Mg^2+^, Mn^2+^, Cd^2+^, Zn^2+^) were not able to keep the substrate bound to the enzyme, only Ca^2+ ^supported the catalytic activity (Table 2). This can be explained by different coordination geometries assumed by the tetrahedral intermediate due to the presence of the Ca^2+ ^ion which not only determines the electrophilic behavior of the catalytic site, but also stabilizes the otherwise flexible Ca^2+^-binding loop and appears to optimize the local protein conformation for substrate interactions [25,26]. Other metals fail to productively bind to the enzyme, competitively inhibit Ca^2+^-mediated activity, or prove to be weakly active [27]. Interestingly Mg^2+ ^and Mn^2+ ^in presence of low Ca^2+ ^concentrations support a significant catalytic activity.

**Table 2:** Effects of different metal ions on the activity of stingray PLA2

Ions	PLA2 Activity (%)	
	
	0 mM Ca^2+^	1 mM Ca^2+^
**Mg^2+^**	3.5 ± 1.1	60 ± 5.4

**Mn^2+^**	2 ± 1	57 ± 2.2

**Zn^2+^**	3 ± 0.5	15 ± 2.4

**Cd^2+^**	2 ± 0.8	12 ± 1.8

Influence of ions concentrations (10 mM each) on SPLA2 activity in the absence or presence of 1 mM Ca^2+ ^was studied using PC emulsion as substrate at 40°C and pH8.5. The control represents 100% of PLA2 activity at 8 mM Ca^2+ ^under the same condition. Values represent the mean of three replicates.

### 3.4. Immunochemical properties

**Figure 2:**
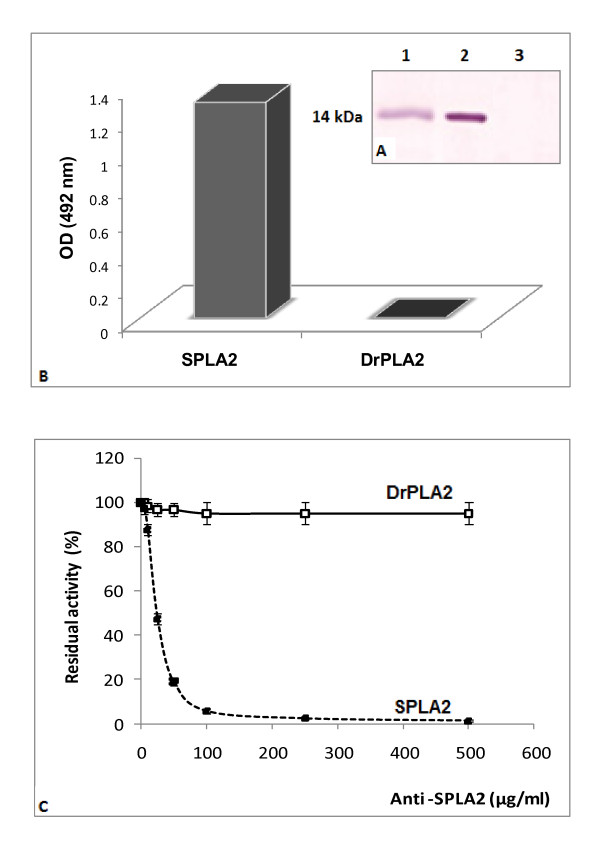
**(A) Immunocross-reactivity of SPLA2 and DrPLA2 with anti- SPLA2 serum using a simple sandwich ELISA**. Each experiment was performed by direct coating of one phospholipase (100 ng/well) with a fixed dilution of 1:500 for the serum tested. **(B) **Immunoblot analysis, SPLA2 solution (200 μg) (lane 1), pure SPLA2 (15 μg) (lane 2) and DrPLA2 (50 μg) (lane 3) using anti-SPLA2 serum at 1:1000 dilution. **(C) **Effect of anti- SPLA2 antibody on PLA2 activity. SPLA2 (12 U) was incubated with various concentrations of anti-SPLA2 in 0.1 M Tris-HC1, 0.1 M NaCl (pH 8.5) for 2 h at 20°C. Similarly, DrPLA2, (12 U) was incubated with anti-SPLA2. After incubation, the remaining activity of PLA2s was measured using PC emulsion in the presence of 4 mM NaTDC and 8 mM CaCl_2_.

The supernatant of the stingray pancreas homogenate containing 200 μg of total proteins was subjected to SDS-PAGE analyses followed by immunoblotting using anti-SPLA2 serum. Our results showed that anti- SPLA2 serum reacted with a single band (14 kDa) corresponding to the SPLA2 present in the crude extract (Figure 2A, lane 1). No other protein bands were recognized by this serum. This result suggests a good specificity of this serum toward SPLA2. Anti-SPLA2 serum was used to carry out cross-reactivities between SPLA2 and DrPLA2 using the ELISA and the Western blotting techniques. SPLA2 was strongly recognized by anti- SPLA2 serum and no cross-immunoreactivity was detected with DrPLA2 (Figure 2A, lanes 1 and 2). For the ELISA technique used for the sake of better sensibility, microtitration plates were coated with a fixed amount of pure SPLA2 and incubated with an antiserum diluted 100 times. Only SPLA2 reacted strongly with its corresponding antiserum (Figure 2B). Figure 2C shows the effect of the anti-SPLA2 antibody on the activities of SPLA2 and DrPLA2. In this experiment, we used an identical PLA2 concentration. The inhibition pattern for marine PLA2 was different from that of mammal one. The anti-SPLA2 antibody, which almost completely inhibited the SPLA2 activity at 0.1 mg/ml, had no effect on the dromedary enzyme. These results might be explained by the fact that SPLA2 may not share common antigenic determinants with classical pancreatic PLA2. Although the 20 residues of SPLA2-NH2-terminal end showed significant homology with those of pancreatic PLA2, the absence of immunoreactivity between DrPLA2 and anti-SPLA2 serum strengthens the idea that SPLA2 could be structurally different from mammalian pancreatic PLA2. This hypothesis needs further structural and biochemical investigations.

### 3.5. Cleavage of SPLA2

**Figure 3:**
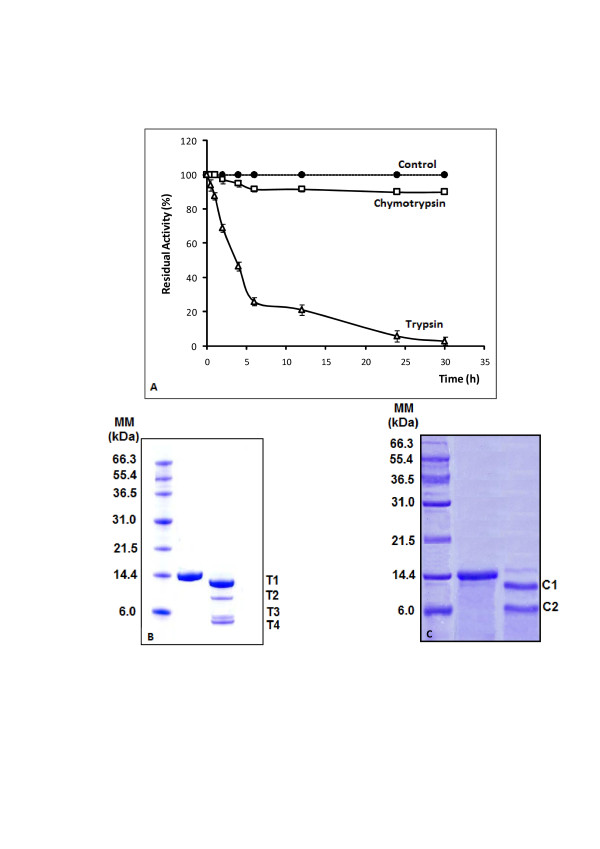
**(A): Remaining activity of SPLA2 incubated with trypsin and chymotrypsin**. SPLA2 was incubated with chymotrypsin or trypsin at 30°C (20:1 w/w) as described in Material and methods section. The remaining activity of SPLA2 cleaved by chymotrypsin and by trypsin towards PC emulsion in the presence of 4 mM NaTDC and 8 mM CaCl_2 _was measured at various incubation times. Samples were withdrawn from the incubation mixtures at various times and analysed. **(B): **SDS-gel electrophoresis analysis of the tryptic cleavage of SPLA2 as a function of time. The gel was stained with Coomassie blue to reveal proteins. Lane 1, molecular mass markers; lane 2, SPLA2; lanes 3, after incubation of SPLA2 with trypsin for 30 h. **(C): **SDS-gel electrophoresis analysis of the chymotryptic cleavage of SPLA2 as a function of time. The gel was stained with Coomassie blue to reveal proteins. Lane 1, molecular mass markers; lane 2, SPLA2; lane 3, after incubation of SPLA2 with chymotrypsin for 24 h.

The SPLA2 incubation with trypsin at 30°C and using a protease/PLA2 molar ratio of 0.05 generated three major fragments: T1 (13 kDa), T2 (8 kDa), T3 (5 kDa) and T4 (4 kDa) with a decrease of PLA2 activity by half within 4 h (Figure 3A and 3B). These fragments were transferred on a PVDF membrane and their N-terminal amino acid was sequenced. The results are given in table 3 together with the corresponding sequences from the PPLA2. The N-terminal sequencing showed that the larger 13 kDa fragment (T1) corresponded to an N-terminal truncated form of SPLA2 starting at residue C11. This N-terminal truncated SPLA2 form (13 kDa) was thus generated from cleavage by trypsin of the K10-C11 bond of SPLA2. The T3 and T4 bands had the same N-terminal sequences as the T1 peptide; whereas The T2 peptide started at residue C66. Based on its molecular mass (7 kDa), this band would correspond to the SPLA2 (14 kDa) lacking N-terminal 65 amino acids. These results suggested that SPLA2 possessed basic residue at position 65 corresponding to R amino acid for PPLA2 and allowing the tryptic cleavage to occur.

**Table 3:** Sequences comparison of protein fragments isolated after chymotryptic and tryptic proteolysis of SPLA2 with PPLA2

For comparison, bold, underlined, and italic symbols of residues indicate identical, homologous, and different amino acids, respectively. The Ca^2+ ^loop (C27-G32) is bold shaded. Stars indicate the amino-acid residues of the catalytic triad. The arrows indicate the tryptic or chymotryptic cleavage site of SPLA2.

Our results showed that the deletion of the N-terminal fragment of SPLA2 (residue A1 to K10) resulted in a loss of the enzyme activity and, thus, provided evidence that the N-terminal fragment was a crucial structural domain necessary for the activity of the enzymes. These findings collaborate with previous findings demonstrating that Helix 1 at the N-terminal of human group 1B PLA2 (hG1B) play an important role in enzyme function. An engineered hG1B lacking the N-terminal helix 1 bound to membranes with weaker affinity exhibited ~100-fold lower enzymatic activity compared with that of the full-length hG1B. It is inferred that this helix 1 facilitates the membrane binding, thus enhances the enzymatic activity based on polarized infrared spectroscopic experiments [28]. Experiments using semi-synthetic hG1B demonstrated that helix 1 residues act as a regulatory domain and mediate interfacial activation [29].

**Figure 4:**
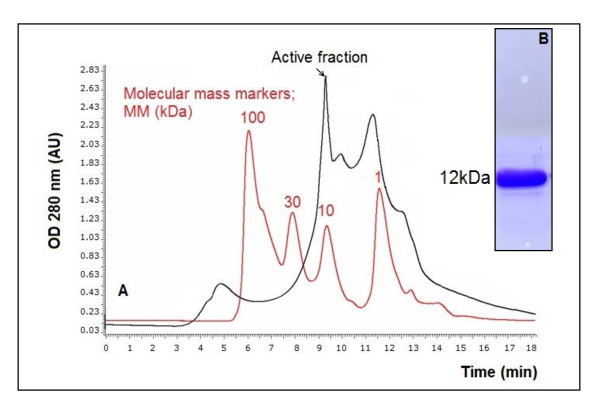
**Filtration on HPLC column and SDS-PAGE (15%) of fragments resulting from chymotryptic cleavage of SPLA2**. **(A) **An aliquot of the incubation mixture (0.1 mg in 0.1 ml) was applied to a HPLC column Bio-sil SEC-125 (300 mm × 7.8 mm) equilibrated in 0.1 M phosphate buffer pH 6.8 containing 0.15 M NaCl, elution was performed at room temperature within 20 min with the same buffer at a flow rate of 1 ml/min. Molecular mass markers were used to estimate the molecular masses of eluted proteins. The effluent was monitored at 280 nm. AU: arbitrary units. **(B) **SDS-PAGE (15%) of the active fraction eluted from HPLC filtration. The gel was stained with Comassie blue to reveal proteins.

Conversely, when incubated with chymotrypsin under the same conditions as described above, the protein band corresponding to the native SPLA2 disappeared within 24 h; whereas 2 major bands (C1, and C2) of different sizes, of about 12, and 7 kDa were accumulated (Figure 4B). The N-terminal sequence of the C1 fragment was the same as the native PLA2. This shows that this 12 kDa fragment is thus issued from a C-terminal truncation of the SPLA-2. The sequencing of the 7kDa fragment showed that it was generated upon cleavage by chymotrypsin of the Y25-G26 bond of SPLA2. Interestingly, the chymotryptic cleavage was not accompanied by any loss of the PLA2 activity assayed on PC emulsion. Furthermore, no C-terminal fragment was detected after proteolysis. The N-terminal C1 fragment alone seemed therefore to be active, despite the absence of the C-terminal fragment.

The 12-kDa form was further purified by filtration on Bio-sil SEC-125 HPLC gel filtration column (300 × 7.8 mm) equilibrated with phosphate 0.1 M buffer pH 7.0 containing 0.15 M NaCl. Elution of proteins was performed with the same buffer at 30 ml/h and SPLA2 emerged 9 min after injection (Figure 4A). The fractions containing the PLA2 activity were pooled, and SDS⁄PAGE analysis revealed only one band corresponding to 12 kDa SPLA2 (Figure 4B).

In order to get more new insights into the mode of protein/substrate interaction, more kinetic behaviour of 12-kDa fragment was checked. The rate of hydrolysis of different concentrations of PC, PE and PS were measured under optimal conditions (4 mM NaTDC, 8 mM, CaCl_2_, and pH 8.5 and 40°C). The Lineweaver-Burk curves were plotted (data not shown). From these fits, the substrate affinity constants (KM) and the turnover of the enzymatic reaction (kcat) were obtained and shown with the deduced catalytic efficiency (kcat/KM) in Table 4. For further comparison, we reported in the same Table 4 the kinetic parameter values obtained with the native SPLA2, and DrPLA2 under the same conditions.

**Table 4:** Apparent kinetic parameters of SPLA2, 12 kDa SPLA2 and DrPLA2

	V_max _(U/mg)			K_m _(mM)			K_cat _(s^-1^)			K_cat_/K_m _(mM^-1 ^s^-1^)		
	
	PE	PC	PS	PE	PC	PS	PE	PC	PS	PE	PC	PS
**SPLA2**	2850 ± 200	750 ± 50	290 ± 15	13 ± 0.7	17.33 ± 0.5	20 ± 1.3	669 ± 5.2	187 ± 2.65	68 ± 2.7	51.5 ± 3.7	11.5 ± 0.5	3.4 ± 0.8

**12 kDa SPLA2**	2730 ± 150	700 ± 80	300 ± 17	13.8 ± 1	16.5 ± 0.9	22 ± 0.7	640.8 ± 7	146.3 ± 3.8	70.42 ± 3.3	47.4 ± 2.2	9.96 ± 0.3	3.2 ± 0.3

**DrPLA2**	2300 ± 120	600 ± 45	190 ± 12	16 ± 1.2	22 ± 0.9	28 ± 1.5	539.9 ± 10	140 ± 2.7	44.6 ± 1.9	33.74 ± 2.8	6.36 ± 0.7	2.03 ± 0.1

From these values, one can say that the two marine PLA2s, showing essentially the same activity toward all the tested substrates, hydrolyse the different phospholipids more efficiently than mammalian one since the ratio representing the catalytic efficiency (Kcat/Km) is about 1.5-2 times higher with native SPLA2 and 12 kDa SPLA2 than with DrPLA2.

**Figure 5:**
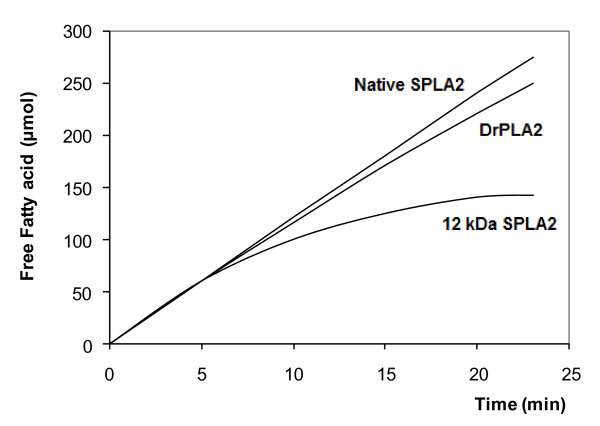
**Kinetics of hydrolysis of PC emulsion by native SPLA2 (12 U), 12 kDa SPLA2 (12 U) or by DrPLA2 (12 U)**. PLA2 activity was followed at pH 8.5 and 40°C in the presence of 4 mM NaTDC and 8 mM CaCl_2_. Each data point represents an average of at least two independent experiments, each in duplicate.

On the other hand, marine and mammalian PLA2 preferentially hydrolyse the phospholipids in the following order PE > PC > PS. Only one unique feature was noted from the activity of 12kDa SPLA2 toward various substrates. The 12 kDa form activity was found to decrease rapidly, in the presence of bile salts and calcium (Figure 5). Under the same conditions, native SPLA2 and DrPLA2 were able to hydrolyze efficiently the different substrates without any denaturation and the kinetics remained linear for more than 25 min (Figure 5). Similar results were obtained when PE or PS were used as substrate (Data not shown).

Several studies have provided evidence that bile salts are tensioactive agents ensuring in their micellar form, the dispersion of the lipolytic products (of hydrolysis) [30,31]. Along the same line, De Haas et al. reported that micellar forms of the substrate were hydrolyzed at a much higher rate than substrates molecularly dispersed by PLA2 [32]. As previously reported SPLA2 and DrPLA2 failed to catalyze the hydrolysis of pure PC. To trigger the PLA2 activity on PC, NaTDC was added prior to the enzyme injection [17,18]. In its presence, the kinetics remained linear for more than 25 min (Figure 5). Similar result was obtained with 12 kDa SPLA2 (data not shown), whereas, the pancreatic chicken PLA2 (ChPLA2) was found to hydrolyze efficiently PC in the absence of NaTDC and its maximum specific activity was found to be nearly independent of NATDC [33]. This difference between SPLA2, DrPLA2 and ChPLA2 might be explained by structural variation of exposed residues between the different phospholipases.

Previous work reported that the overall structural and functional perturbations caused by deleting nine C-terminus residues of bovine pancreatic PLA2 over expressed in Escherichia coli were modest, but the C-terminus deletion mutant displayed an interesting and significant property. It functioned well at the anionic interface, but its activity decreased substantially at the zwitterionic interface possibly due to the uncoupling between calcium binding and substrate (inhibitor) binding [34].

## 4. Conclusion

Marine and mammal pancreatic PLA2 share a high amino-acid sequence homology. However; the absence of cross-immunoreactivity between DrPLA2, taken as mammal model, and anti-SPLA2 serum strengthens the idea that SPLA2 could be structurally different from mammalian pancreatic PLA2. Further investigations are needed to better establish the structure-function relationship of this class of enzyme and to identify key residues involved in substrate recognition responsible for biochemical differences between the classes of PLA2.

## Competing interests

The authors declare that they have no competing interests.

## Authors' contributions

ABB: designed the study and drafted the manuscript.

HM: helped to draft the manuscript and participated in the design of the study.

All authors read and approved the final manuscript.
